# Vinorelbine as substitute for vincristine in patients with diffuse large B cell lymphoma and vincristine-induced neuropathy

**DOI:** 10.1007/s00520-021-06059-2

**Published:** 2021-02-24

**Authors:** Stefan Hatzl, Florian Posch, Arwin Rezai, Maximilian Gornicec, Christine Beham-Schmid, Theresa Magnes, Sandro Wangner, Alexander Deutsch, Hildegard Greinix, Barbara Uhl, Katharina T. Prochazka, Alexander Egle, Richard Greil, Thomas Melchardt, Werner Linkesch, Eduard Schulz, Peter Neumeister

**Affiliations:** 1 Division of Hematology, Department of Internal Medicine, Medical University of Graz, Graz, Austria; 2 Division of Oncology, Department of Internal Medicine, Medical University of Graz, Graz, Austria; 3 Laboratory for Immunological and Molecular Cancer Research, Oncologic Centre, 3rd Medical Department with Hematology and Medical Oncology, Hemostaseology, Rheumatology and Infectious Diseases, Paracelsus Medical University Salzburg, Salzburg, Austria; 4 Institute of Pathology, Medical University of Graz, Graz, Austria

**Keywords:** Vino-R-CAP, Peripheral neuropathy, DLBCL, R-CHOP, Vinorelbine

## Abstract

**Background:**

A combination of rituximab with cyclophosphamide, doxorubicin, vincristine, and prednisone (R-CHOP) is the standard first-line therapy for diffuse large B cell lymphoma (DLBCL), the most common aggressive lymphoma in adults. One of the major adverse effects of this regimen is vincristine-induced polyneuropathy which leads to discontinuation of vincristine in up to 30% of DLBCL-patients. Dose reduction of vincristine might worsen treatment outcomes of DLBCL but identification of treatment alternatives for patients exhibiting peripheral neuropathy during R-CHOP is an unmet need in hematology.

**Methods:**

In this retrospective cohort study, comprising 987 patients with de novo DLBCL, we delineated the role of vinorelbine as a substitute for vincristine in R-CHOP by measuring improvements in neuropathy and outcome variables.

**Results:**

Five-year overall survival (OS) and progression-free survival (PFS) were 72.6% and 63.1% in patients who received regular doses of vincristine, as compared to 60.6% and 51.7% in patients who received reduced doses of vincristine (*p* = 0.022 and *p* = 0.003, respectively). Of 199 patients who switched to vinorelbine, the majority experienced an improvement of neuropathy Furthermore, vinorelbine-switched patients showed favorable oncologic outcomes.

**Conclusion:**

Replacement of vincristine by vinorelbine due to neuropathy is effective and safe, and results in a significant improvement in neuropathy as compared to treatment with R-CHOP.

**Supplementary Information:**

The online version contains supplementary material available at 10.1007/s00520-021-06059-2.

## Introduction

Diffuse large B cell lymphoma (DLBCL) is the most common aggressive non-Hodgkin lymphoma (NHL) in adults accounting for more than 30% of all NHL cases [1, 2]. Vincristine, a microtubule assembly inhibitor, has become an integral component of today’s treatment of DLBCL since its discovery as an active agent against lymphoma. A combination of vincristine with cyclophosphamide, hydroxydaunorubicin, prednisone, and the monoclonal antibody rituximab (R-CHOP) administered in 21-day intervals is the standard of care and results in 5-year overall survival (OS) rates of up to 87% depending on risk factors [3, 4]. However, all clinical trials investigating the addition of novel agents to the R-CHOP backbone as first-line treatment failed to show improvement of OS so far [5].

Vincristine-induced peripheral neuropathy (VIPN), which is commonly a sensorimotor neuropathy, remains a major complication of lymphoma patients treated with R-CHOP. VIPN can be observed in 20–40% of DLBCL patients leading to an increase in morbidity and a decrease in quality of life [6–9]. However, the clinical features and symptoms are variable and range from mild neurologic signs like numbness, which may disappear with discontinuation of vincristine, to severe permanent neurological long-term sequela such as gross and fine motoric deficits, e.g., impaired handwriting [10, 11].

The key mechanism of vinca-alkaloid action and induction of neuropathy is inhibition of microtubule polymerization but not all vinca-alkaloids cause the same rate and intensity of chemotherapy-induced neuropathy [12]. Vincristine is the most neurotoxic vinca-alkaloid resulting in peripheral neuropathy of any grade in 18 to 70% of adult patients [13, 14]. On the contrary, the vinca-alkaloid vinorelbine is rarely associated with peripheral neuropathy occurring in only about 4% of patients [15].

Dose reduction or discontinuation of vincristine has been commonly used to ameliorate VIPN in R-CHOP-treated DLBCL patients, although this practice is likely associated with impaired treatment outcomes and higher lymphoma-related mortality [16–20]. Nevertheless, there is a lack of studies focusing explicitly on VIPN and the consequences of vincristine dose reductions. In this cohort study, we aimed to delineate the role of vinorelbine as a replacement for vincristine in R-CHOP after onset of peripheral neuropathy in the treatment of de novo DLBCL.

## Patients and methods

### Cohort description

**Fig. 1:**
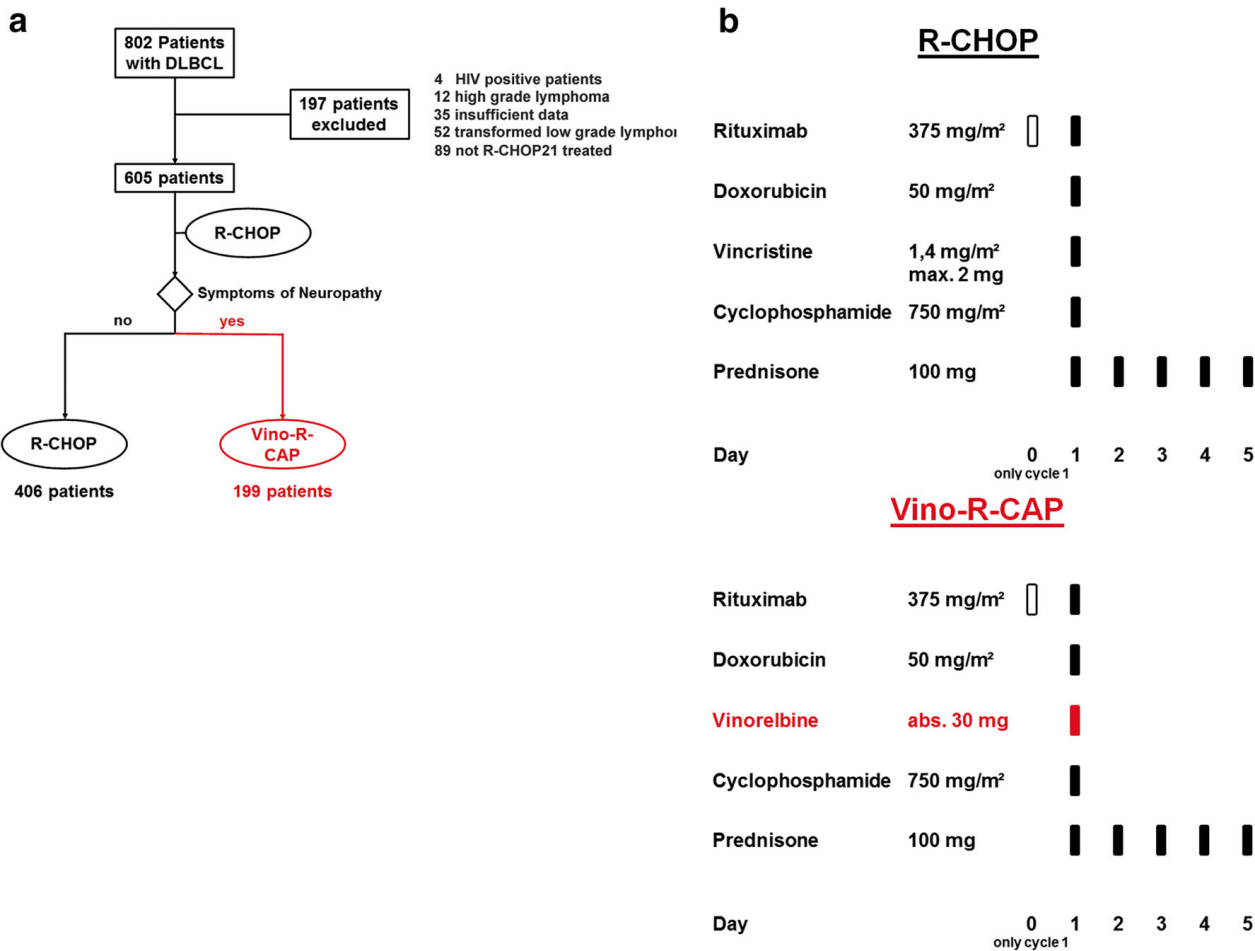
Full trial protocol of the “Graz cohort” (*n* = 605). **a** Flow diagram: Comparison of patients which underwent R-CHOP or Vino-R-CAP treatments. **b** Description of Vino-R-CAP and R-CHOP treatment regimens administered in the cohort. Abbreviations: Vino-R-CAP vinorelbine, rituximab, cyclophosphamide, hydroxydaunorubicin, prednisone; R-CHOP rituximab, cyclophosphamide, hydroxydaunorubicin, vincristine, prednisone

In this retrospective bi-center cohort study, we included 987 patients who were presented for treatment of histologically confirmed de novo DLBCL at the Division of Hematology, Medical University of Graz (“Graz cohort”: *n* = 605), or the third Medical Department at the Paracelsus Medical University Salzburg (“Salzburg cohort”: *n* = 382), both of which are located in Austria, between 2001 and 2020. Patients with human immunodeficiency virus (HIV) positivity, transformed low-grade lymphoma, high-grade lymphoma, and Burkitt lymphoma, and patients who did not receive full dosage R-CHOP in 21-day cycles (R-CHOP21) were excluded in the Graz cohort for evaluation of vinorelbine treatment (Fig. 1a). Patients treated within the Salzburg cohort met the same inclusion criteria, but dosage of vincristine and doxorubicin was adapted according to adverse events (Supplementary Table 1). Data on baseline characteristics and clinical outcomes were ascertained from our in-house electronic healthcare database system, as previously described [21, 22]. The research project was approved by the local institutional review boards (EK-Votum: 32-306 ex19/20 ethikkommission@medunigraz.at and EK-Votum: 415-EP/73/127-2012 ethikkommission@salzburg.gv.at).

### Rationale behind switching to vinorelbine

Replacement of vincristine in R-CHOP by vinorelbine has been established as a local standard on an empirical basis in Graz to deliver the full antitumor activity of a vinca-alkaloid after development of any sign of neuropathy. The rationale behind this approach is the substitution of the most neurotoxic vinca-alkaloid vincristine with one of the least neurotoxic, i.e., vinorelbine, in a regimen called Vino-R-CAP in order to reduce or stop progression of VIPN without discontinuation of the substance class.

### Treatment and local standard for switching to Vino-R-CAP

Patients without any signs of neuropathy were treated with R-CHOP and received the combination of 375 mg rituximab per square meter of body-surface area, on day 0 of the treatment cycle; 750 mg of cyclophosphamide per square meter on day 1; 50 mg of doxorubicin per square meter on day 1; 1.4 mg of vincristine per square meter, up to a maximal dose of 2 mg, on day 1; and 100 mg of prednisone per day for 5 days. They were treated every 3 weeks for six to eight cycles of R-CHOP [23]. Patients who developed neutropenia grade 4 (< 500 cells/μL (0.5 × 10^9^ cells/L)) or febrile neutropenia after any cycle of chemotherapy received prophylactically granulocyte colony-stimulating factor.

Patients who developed any sign of neuropathy during R-CHOP treatment were switched to Vino-R-CAP during the next cycle and received 30 mg of vinorelbine absolute, on day 1 instead of vincristine (Fig. 1a and b).

### Assessment of chemotherapy-induced neuropathy

We assessed VIPN using the National Cancer Institute Common Terminology Criteria for Adverse Events (NCI-CTCAE) neuropathy sensory subscale version 3. This outcome measure can be easily accessed by clinicians and used to score patients’ symptoms from 0 to 4 (no symptoms to life-threatening symptoms). However, the measure has also received some criticism due to low interobserver reliability, underestimation of symptoms, and reproducibility of this measure [24–26]. To further improve the reliability of the neuropathy assessment, we included a second, more accurate, and reproducible measurement, the clinical Total Neuropathy Score (cTNS) which is validated for VIPN [27–29]. It comprises seven different parameters of neuropathy (sensory symptoms, motor symptoms, autonomic symptoms, pin sensibility, vibration sensibility, strength, and tendon reflexes) and ranges from 0 to 24 with a higher total score correlating with increasing the severity of the neuropathy [30–32].

### Outcomes

For time-to-event analyses of oncologic outcome, we considered three outcomes: overall survival (OS), progression-free survival (PFS), and DLBCL-specific survival. These outcomes were defined as the time from the date of diagnosis until the occurrence of death-from-any-cause or censoring alive (OS), disease progression or death-from-any-cause or censoring alive, whatever came first (PFS), or death-related-to-DLBCL or censoring alive or due to death-from-other-causes, whatever came first, respectively. To conduct neuropathy analyses, we considered the continuous change in NCI CTCAE version 3.0 neuropathy grading [27, 30], the cTNS grade, and cTNS score [29] from switching to vinorelbine until 2 months thereafter. Moreover, we calculated the proportions of patients who displayed worsening, stable, and improving neuropathy symptoms according to these scores.

### Statistical methods

All statistical analyses were performed with Stata (Windows version 15.1, Stata Corp., Houston, TX, USA). Continuous data were reported as medians [25th to 75th percentile], and the count data were reported as absolute frequencies (%). Correlations were computed with Spearman’s rank-based rho, and associations between variables were investigated with rank-sum tests, χ^2^ tests, and Fisher’s exact tests, simple or multiple linear regression, and paired *t* tests, as appropriate. The median follow-up was estimated with the reverse Kaplan-Meier estimator. OS, PFS, and DLBCL-related survival were computed with Kaplan-Meier estimators and compared between two or more groups with log-rank tests. Risks of relapse, death-related-to-DLBCL, and death-from-other-causes were estimated with competing risk cumulative incidence estimators. Modelling of OS, PFS, and DLBCL-related hazard functions was performed with uni- and multivariable Cox regression analyses. A *p* value of < 0.05 was considered statistically significant. Importantly, to eliminate immortal time bias, the switch from R-CHOP to Vino-R-CAP was modelled as a so-called time-dependent variable within Cox models. This was achieved by partitioning the follow-up time of patients who were switched to vinorelbine into times before and after the replacement. For visual display of the association between the replacement of vincristine by vinorelbine and oncologic outcomes, we performed landmark analyses after the fourth cycle. This landmark date was chosen because most restaging investigations of DLBCL were performed after the fourth immunochemotherapy cycle according to local standards. In subgroup analyses, we fitted interactions between the switch to vinorelbine as time-dependent variables and selected prognostically relevant co-variables of interest. Interaction *p* values < 0.05 were considered to indicate statistical significance in these sub-analyses. Missing data are reported in Table 1, and a complete case analysis was performed. The full analysis code is available on request from FP.

**Table 1:** Baseline characteristics of the study population: distribution by switch status (*n* = 605).

Variable	*N* (% miss.)	Overall (*n* = 605)	Vincristine group (*n* = 406)	Vinorelbine group (*n* = 199)	*p* value*
Demographic variables					
Age (years)	605 (0%)	65 [54–74]	64 [52–74]	65 [56–73]	0.835
Female gender	605 (0%)	299 (49%)	197 (49%)	102 (51%)	0.527
ECOG (points)	604 (0%)	1 [0–1]	1 [0–1]	1 [0-1]	0.106
Tumor characteristics					
Cell of origin: Non-GCB	575 (5%)	300 (52%)	212 (53%)	88 (50%)	0.488
Clinical stage: III–IV	605 (0%)	322 (53%)	209 (51%)	113 (57%)	0.219
Extranodal manifestation	605 (0%)	320 (53%)	216 (53%)	104 (52%)	0.828
Double expressor biology**	405 (33%)	/	/	/	0.253
0 points	/	79 (20%)	59 (20%)	20 (19%)	/
1 point	/	231 (57%)	176 (59%)	55 (52%)	/
2 points	/	95 (23%)	64 (21%)	31 (29%)	/
Risk stratification					
R-IPI (points)	605 (0%)	2 [2–3]	2 [2–3]	3 [2–3]	0.491
NCCN-IPI (points)	605 (0%)	3 [2–5]	3 [2–4]	3 [2–5]	0.269
CNS-IPI (points)	605 (0%)	1 [1–2]	1 [1–2]	2 [1–2]	0.239
Treatment characteristics					
Cycles of primary treatment	605 (0%)	6 [6–8]	6 [6–8]	6 [6–8]	**0.005**
Cycles before switch to vinorelbine	199 (0%)	/	/	2 [2–4]	**/**
ASCT	605 (0%)	45 (7%)	29 (7%)	16 (8%)	0.693
Outcomes					
Best response to first-line therapy	605 (0%)	/	/	/	**0.009**
CR	/	498 (82%)	322 (79%)	176 (88%)	/
PR	/	46 (8%)	33 (8%)	13 (7%)	/
PD	/	61 (10%)	51 (13%)	10 (5%)	/
Long-term outcomes	605 (0%)	/	/	/	**0.004**
No relapse	/	422 (70%)	269 (66%)	153 (77%)	/
Relapse	/	87 (14%)	59 (15%)	28 (14%)	/
Primary progression	/	96 (16%)	78 (19%)	18 (9%)	/
Death from DLBCL	605 (0%)	144 (24%)	105 (26%)	39 (20%)	0.089
Death from other causes	605 (0%)	107 (18%)	65 (16%)	42 (21%)	0.123

*N* (% miss.) denotes the number of patients with fully observed variable (% missing denotes the percentage of patients with lack of data). *p values are from rank-sum tests, χ^2^ tests, and Fisher’s exact tests, as appropriate. **Double expressor biology as assessed by immunohistochemistry for BCL2 and MYC with scoring according to the published algorithm of Hans et al. [33]

*Abbreviations*: *ECOG* Eastern Cooperative Oncology Group performance status, *GCB* germinal center B cell, *R-IPI* Revised International Prognostic Index, *NCCN-IPI* National Comprehensive Cancer Network International Prognostic Index, *CNS-IPI* Central Nervous System International Prognostic Index, *ASCT* autologous stem cell transplantation, *CR* complete remission, *PR* partial remission, *PD* progressive disease, *DLBCL* diffuse large B cell lymphoma

## Results

### Evaluation of treatment outcomes after reduction of vincristine due to neuropathy

**Fig. 2:**
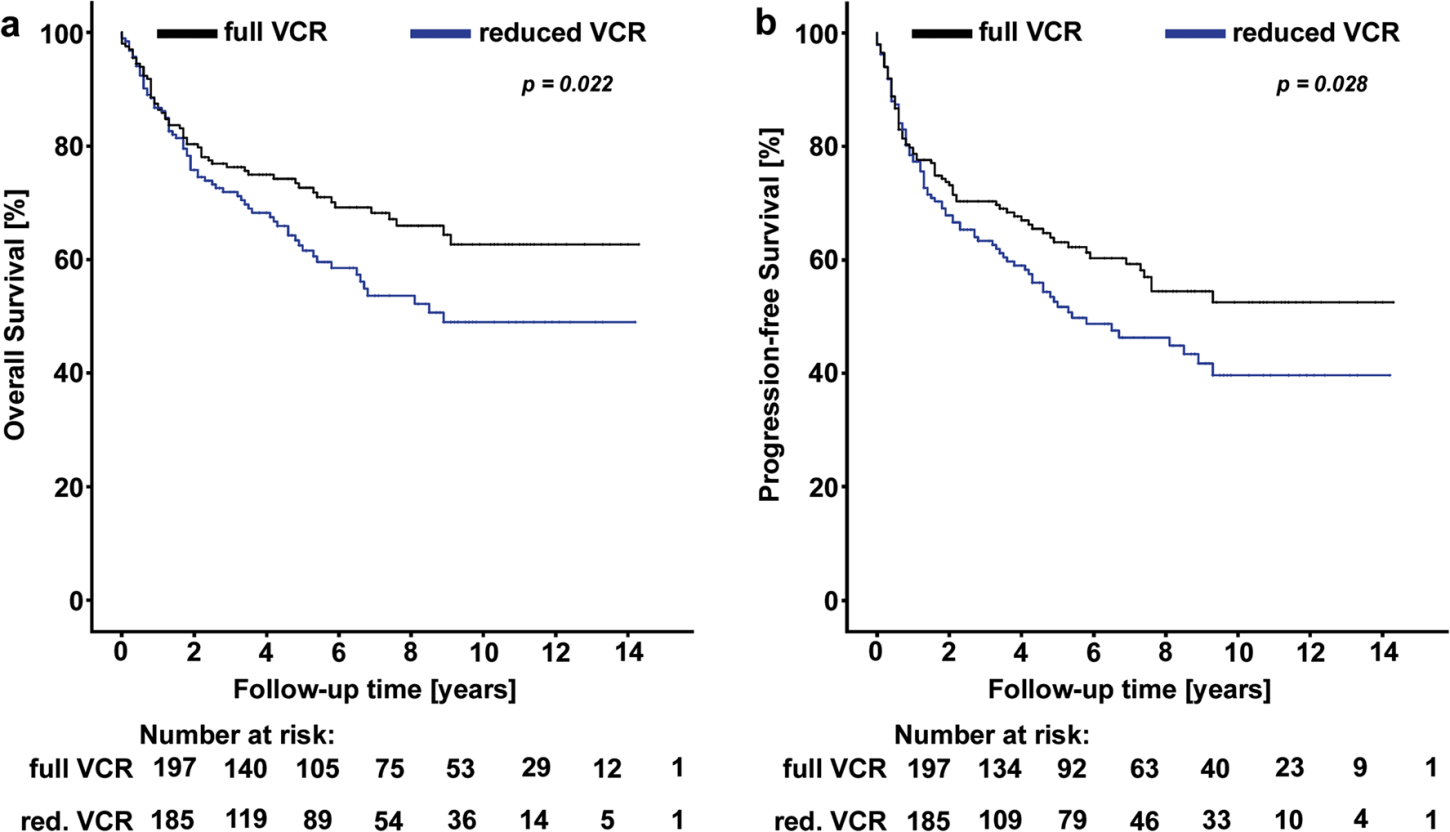
Survival analyses of oncologic outcome according to vincristine dose reduction in the “Salzburg cohort” (*n* = 382). **a** Overall survival. **b** Progression-free survival. Abbreviations: VCR vincristine

First, we clarified whether the standard of care (dose reduction or discontinuation of vincristine due to neuropathy) led to impaired outcomes. Therefore, we analyzed 382 DLBCL patients who were treated with R-CHOP (“Salzburg cohort”). In these patients, the vincristine dose was reduced to 1 mg or discontinued if neuropathic signs were observed during the treatment course at the discretion of the treating physician (Supplementary Table 1). In this cohort, the 1-, 5-, and 10-year PFS rates were 78.0% (95% CI, 74.1–82.6), 57.6% (52.4–62.4), and 46.2% (28.2-65.5), respectively. The corresponding 1-, 5-, and 10-year OS rates were 87.1% (83.6–90.1), 67.3% (62.3–73.1), and 56.2 % (33.6–78.3), respectively. The Kaplan–Meier analysis results revealed a highly significant association between vincristine dose reduction and poor OS (Fig. 2a) and PFS (Fig. 2b). In detail, 5-year OS and PFS were 72.6% (95% CI, 75.9–69.1) and 63.1% (58.3–68.0) in patients with full dosage of vincristine during the whole treatment course, and 60.6% (64.4–66.5) and 51.7% (41.1–61.0%) in patients receiving reduced dose vincristine (log-rank *p* = 0.022 and *p* = 0.028 for OS and PFS, respectively). To determine the independent prognostic value of vincristine dose reduction, multivariable analyses were carried out including R-IPI, ECOG, and doxorubicin dose reduction (Supplementary Table 2). Here, the prognostic association between vincristine dose reduction and worse OS and PFS prevailed.

### Baseline characteristics and treatment outcomes of patients treated with Vino-R-CAP

Having shown that dose reduction or discontinuation of vincristine results in impaired clinical outcomes, we next investigated the effects of replacing vincristine with vinorelbine because of VIPN. The “Graz cohort” consisted of 605 patients (median age, 65 years; female, 49%; median NCCN-IPI, 3 points; Table 1) and approximately half of the cohort had clinical stage III–IV disease, extranodal manifestation, and/or a non-germinal center B cell (GCB) cell of origin tumor. The overall response rate to first-line therapy was 90% (95%CI, 87–92). During a median follow-up of 8.5 years, we observed 96 primary disease progressions (16%), 87 relapses (14%), 144 deaths from DLBCL (24%), and 107 deaths from other causes (18%), respectively. This corresponded to 1-, 3-, 5-, and 10-year OS estimates of 88%, 75%, 70%, and 54%, respectively. The corresponding PFS estimates and risks of relapse, death-from-lymphoma, and death-from-other-causes are reported in Supplementary Figure 1 and Supplementary Table 3.

One hundred and ninety-nine patients (33%) were switched from R-CHOP to Vino-R-CAP after a median of two cycles [25th–75th percentile, 2–4] due to VIPN. Baseline characteristics were similar between both groups (Table 1), except that patients who were switched to Vino-R-CAP received on average 0.45 cycles more therapy (*p* = 0.005) which was related to a slightly lower prevalence of primary progressive disease and thus a higher likelihood of competing first-line therapy (*p* = 0.009). This association between change of therapy and slightly higher number of treatment cycles did not prevail after adjusting for treatment response (Supplementary Table 4).

### Neuropathy improves after switching from R-CHOP to Vino-R-CAP

Next, we determined whether switching from vincristine to vinorelbine could improve neuropathy symptoms as measured by NCI-CTCAE and cTNS.

**Fig. 3:**
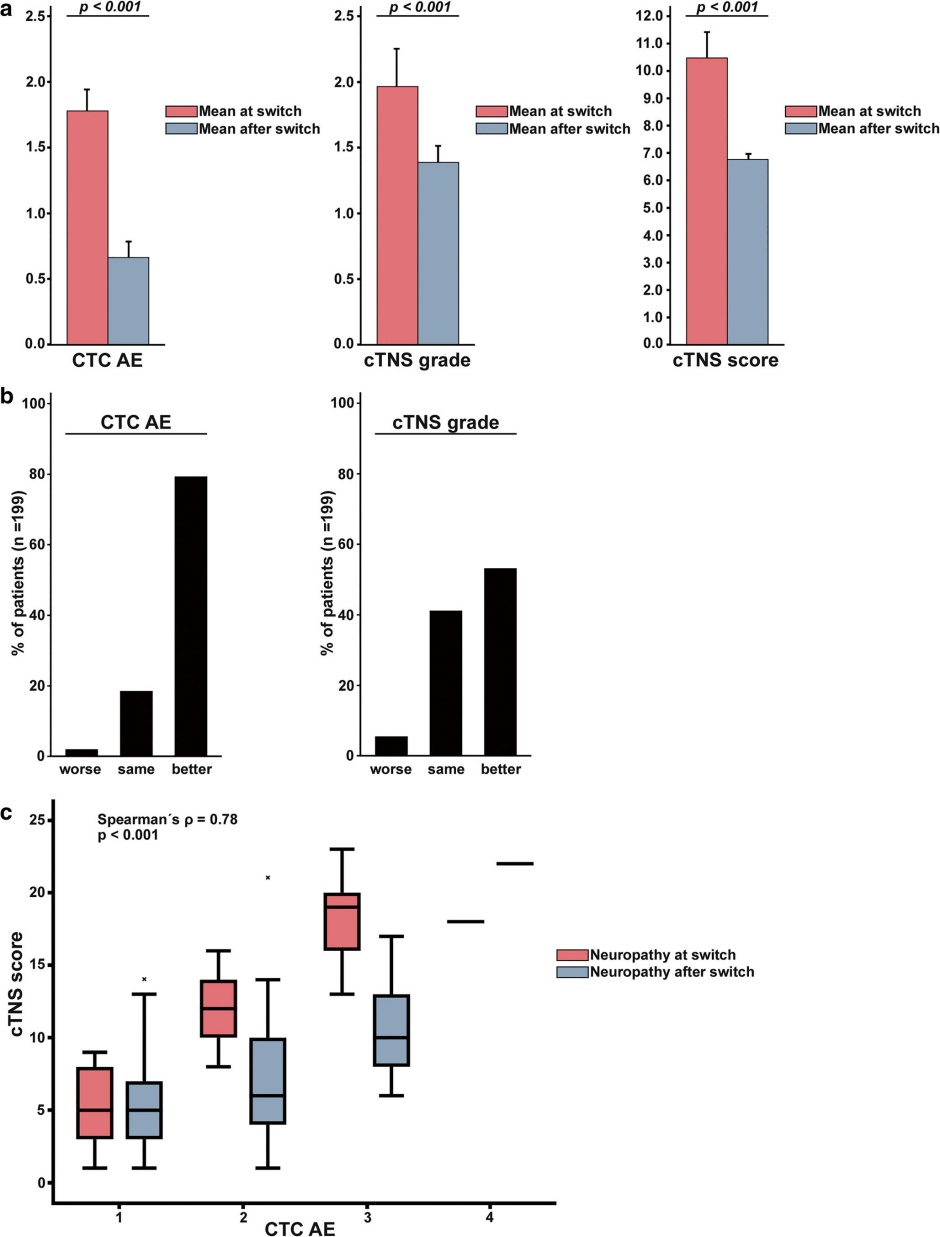
Two measures of neuropathy in patients who were switched to vinorelbine (*n* = 199). **a** Mean neuropathy scores at and after switch to vinorelbine. **b** Proportion of patients with worsening, stable, and improving neuropathy after switching to vinorelbine. **c** Correlation between neuropathy grades according to NCI CTC and TNS scores

In the 199 patients who changed over to Vino-R-CAP, neuropathy severity at the time of the switch according to NCI-CTCAE was assessed as grade 1 in 63 patients (32%), grade 2 in 118 patients (59%), grade 3 in 17 patients (9%), and grade 4 in one patient (1%). This patient with grade 4 neuropathy had to be admitted to the neurologic intensive care unit due to progressive paralysis. Measures of neuropathy severity improved from the time the switch occurred until 2 months after the last treatment cycle. In detail, neuropathy according to NCI-CTCAE improved by 1.1 grades, and neuropathy according to the cTNS grade by 0.6 grades (both *p* < 0.0001, Fig. 3a and Supplementary Table 5). Similarly, neuropathy according to NCI-CTCAE and cTNS improved in 79% and 53% of patients, respectively, with only 2% and 6% of patients displaying worsening neuropathy on the Vino-R-CAP regimen (Fig. 3b). The two neuropathy scoring systems were highly correlated with each other (Spearman’s *ρ* correlating neuropathy scores at switching according to NCI-CTCAE and TNS grade=0.78, *p* < 0.001, Fig. 3c). These results demonstrate a favorable neuropathy outcome after replacing vincristine with vinorelbine.

### Switching from R-CHOP to Vino-R-CAP is associated with a higher survival outcome

**Fig. 4:**
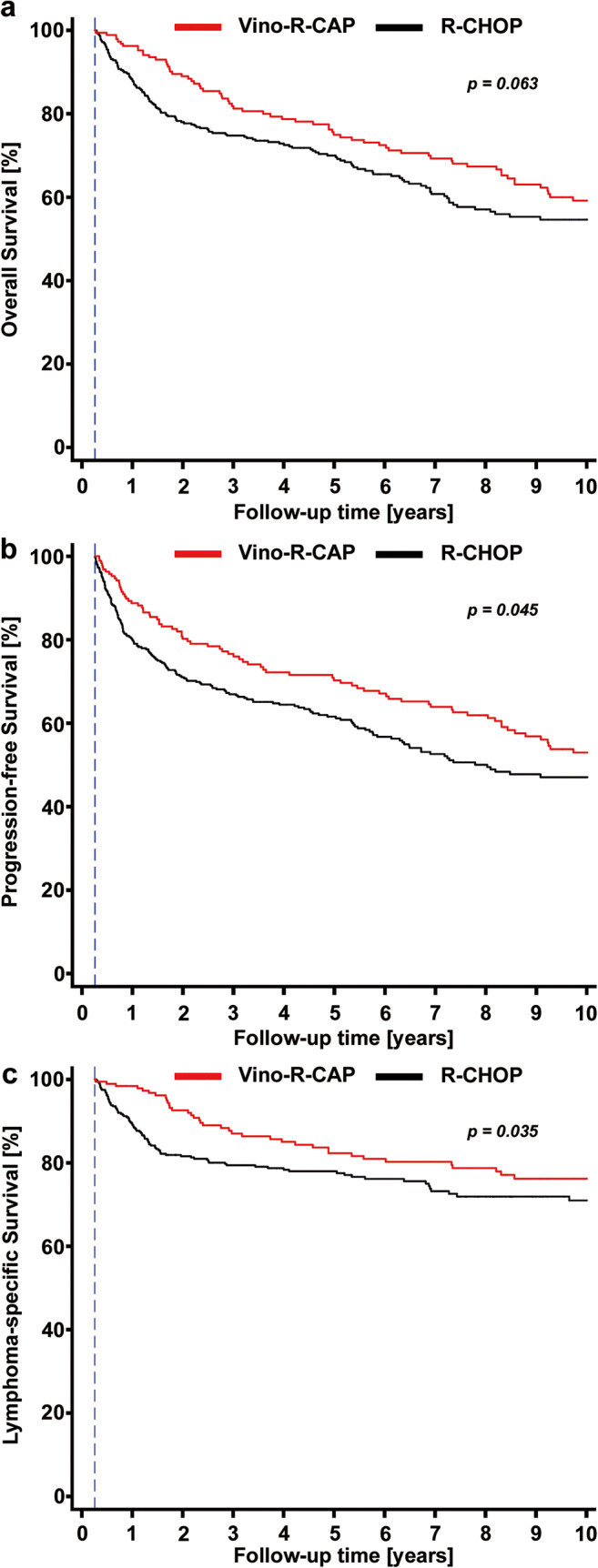
Landmark analyses of oncologic outcome according to switch to vinorelbine. The landmark date (blue dashed line) was set at the fourth treatment cycle (approximately 94 days of follow-up). **a** Overall survival. **b** Progression-free survival. **c** DLBCL-specific survival

**Fig. 5:**
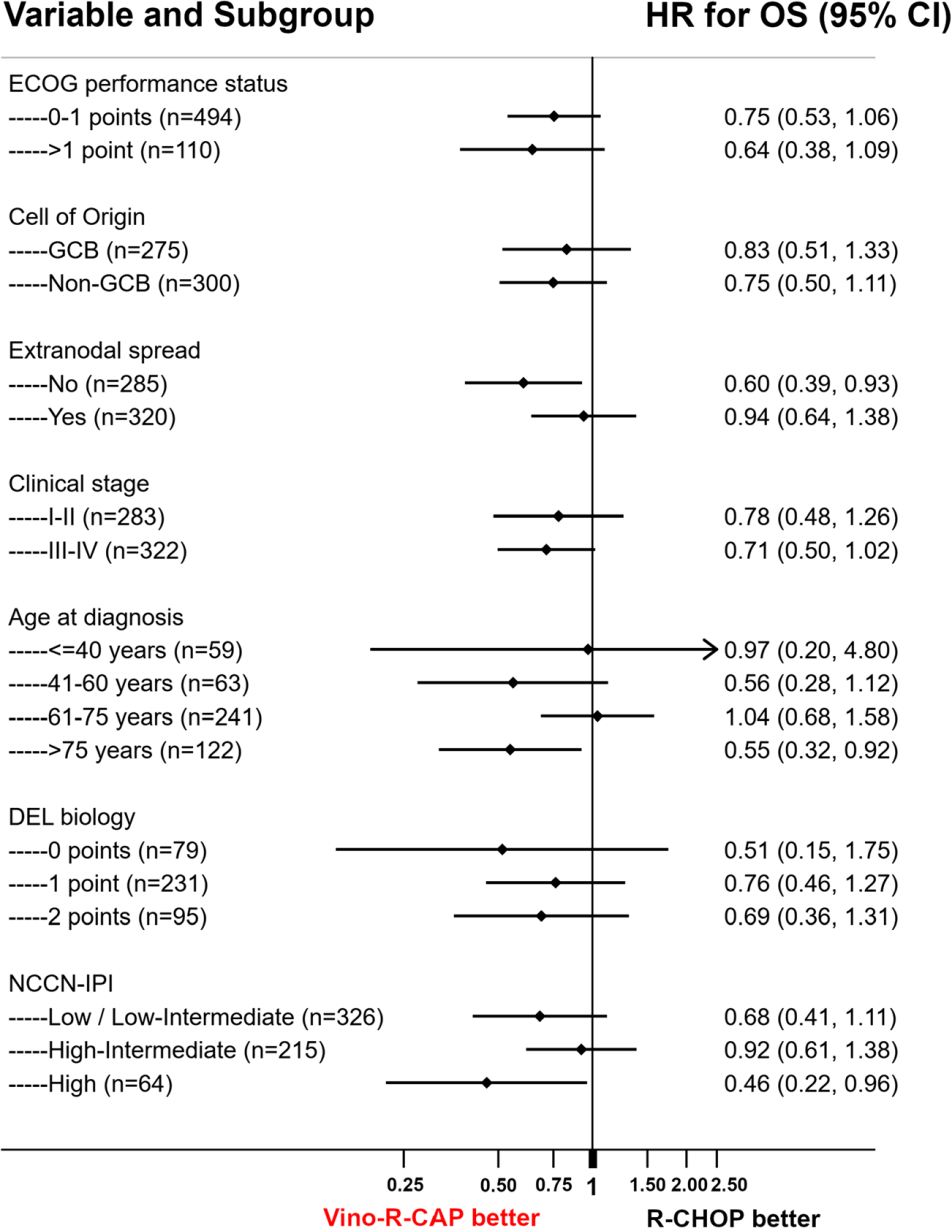
Subgroup analysis: Forest plot of the relative association of R-CHOP and Vino-R-CAP with 10-year overall survival according to selected clinical co-variables. Black dots represent the subgroup hazard ratio, and the associated bars the 95% confidence interval. The black vertical line represents the “line of unity,” at which patients who were and were not switched to vinorelbine have similar hazards of death-from-any-cause. Regression results were obtained by fitting an interaction between the vinorelbine switch as a time-dependent variable and the respective subgroup variable. Note that the “Low” and “Low-Intermediate” categories of the NCCN-IPI had to be merged due to an extremely low number of deaths in the “Low” group that prevented convergence of the regression model. Abbreviations: HR hazard ratio, OS overall survival, 95%CI 95% confidence interval, ECOG Eastern Cooperative Oncology Group, GCB germinal center B cell, DEL double expressor lymphoma, NCCN-IPI National Comprehensive Cancer Network International Prognostic Index

To further evaluate outcomes in patients treated with Vino-R-CAP, we performed a univariable time-to-event regression analysis treating substitution of vincristine as a time-dependent variable. Patients who changed over to Vino-R-CAP displayed better outcomes regarding OS, PFS, and DLBCL-related mortality (Supplementary Table 6). These findings prevailed in multivariable regression analyses adjusting for important prognostic co-variates, such as NCCN-IPI, double expressor biology, and cell of origin (Supplementary Table 6). In a landmark analysis after the fourth treatment cycle (94 days of follow-up), 5-year OS estimates were 75% and 70% in patients that were and were not switched over to vinorelbine (Mantel-Byar *p* = 0.063, Fig. 4a). Accordingly, PFS and DLBCL-related survival were significantly in favor of Vino-R-CAP. Compared to R-CHOP, 5-year PFS estimates were 73% versus 62% (Mantel-Byar *p* = 0.045), and DLBCL-related survival estimates 84% versus 78% (Mantel-Byar *p* = 0.035), respectively (Fig. 4b, c). Subgroup analyses were performed to determine which patients benefited the most from switching to Vino-R-CAP. Particularly favorable associations between Vino-R-CAP and long-term survival outcome were identified in elderly patients, in patients who crossed over to Vino-R-CAP after the first three cycles of R-CHOP therapy, and in patients with high-risk DLBCL according to NCCN-IPI (Supplementary Table 8, Fig. 5). Overall, the favorable long-term outcome of patients who switched to Vino-R-CAP was consistent across all subgroups such as ECOG performance status (interaction *p* = 0.619), clinical stage (interaction *p* = 0.709), extranodal disease manifestation (interaction *p* = 0.131), and double expressor lymphoma biology (interaction *p* = 0.839, Fig. 5).

### R-CHOP and Vino-R-CAP show similar adverse events

Adverse events during Vino-R-CAP treatment were consistent with the expected toxic effects of R-CHOP occurring with similar frequencies in both groups (Supplementary Table 7) [6]. The most common adverse event was infection which occurred in 48% of R-CHOP and 46% of Vino-R-CAP treated patients. Accordingly, there was no difference in severe chemotherapy-related events like neutropenia, thrombocytopenia, or organ toxicity.

## Discussion

The R-CHOP immunochemotherapy regimen achieves reasonable survival and cure rates in patients with DLBCL [4, 6, 34]. Despite all efforts which have been made in unraveling the molecular mechanisms of lymphomagenesis, establishing new risk models for outcome prediction, and employing promising new substances for treatment within clinical trials, (R)-CHOP continues to represent the standard first-line therapy for the last 40 years [2]. One of the major toxic sequelae caused by this regimen is VIPN which affects between 20 and 40% of all patients [4, 6–9]. A retrospective analysis from Korea reports VIPN rates of up to 85% in patients with DLBCL or follicular lymphoma treated with R-CHOP causing deterioration in quality of life [35]. Known risk factors of VIPN are cumulative dose of vincristine, older age, and ethnicity, as well as genetic polymorphisms [36–39]. The most common approach to influence VIPN during R-CHOP treatment is dose reduction or discontinuation of vincristine. However, there is increasing evidence that dose-dense chemotherapy is seminal for improving remission and survival rates [16–20]. To the best of our knowledge, there are only two published studies focusing on the issue of vincristine dose reduction in DLBCL patients delivering contradictory results regarding survival outcomes [19, 40]. Utsu et al. showed significantly impaired outcomes in patients with vincristine dose reduction due to neuropathy which is in agreement with our data provided in the “Salzburg-Cohort.” They had very stringent inclusion criteria and considered only de novo DLBCL patients treated with R-CHOP21 similar to our study [19]. On the contrary, there was no difference in treatment outcomes between patients with or without vincristine dose reduction in the study by Mörth et al. However, they had more arbitrary inclusion criteria with a more heterogeneous patient population and different chemotherapy regimens probably leading to a profound selection bias. Nonetheless, both studies did not demonstrate an improvement of VIPN after vincristine dose reduction.

Here, we present a large “real world” cohort of patients with de novo DLBCL treated with R-CHOP in 21-day cycles showing a VIPN rate of 32% (199/605) which is in agreement with published large prospective trials [4, 6–9]. We provide for the first time evidence of a neurologic benefit following substitution instead of dose reduction of vincristine. This benefit was documented by two different neuropathy measures, the easily assessable NCI-CTCAE score and the more accurate and reliable cTNS, which correlated significantly with each other [27–29]. Furthermore, our results suggest an improvement of survival outcomes after switching to Vino-R-CAP. One might speculate that patients who were treated with vinorelbine during first-line treatment received an additional cytostatic substance targeting remaining vincristine-resistant lymphoma cells [15, 41]. It has been established that vinorelbine is an effective salvage agent in relapsed or refractory aggressive NHL. Blazarotti et al. showed overall response rates of 46% for vinorelbine as single agent in heavily pre-treated lymphoma patients [42]. Moreover, vinorelbine has been used in different salvage regimens for relapsed or refractory DLBCL as combination partner because it has no-cross resistance properties with other vinca alkaloids [43, 44]. However, these findings are preliminary and can only be interpreted as hypothesis generating.

The main limitation of our study is its retrospective nature; however, the patient cohort is characterized by very stringent inclusion criteria and long follow-up providing a 20-year “real world” experience. Although it has to be proven in a randomized controlled trial, based on our robust retrospective data, we can conclude that switching to Vino-R-CAP is safe and more effective than reducing vincristine dose when DLBCL patients develop neuropathy during treatment with R-CHOP.

## Supplementary Information

ESM 1 (DOCX 88 kb)

## Data Availability

The full dataset is available on request from SH.

## Code availability

The full analysis code is available on request from FP or SH.
