# Supplementary material for: Vinorelbine as substitute for vincristine in patients with diffuse large B cell lymphoma and vincristine-induced neuropathy
Source: Support Care Cancer. 2021 Feb 24;29(9):5197–207. doi: 10.1007/s00520-021-06059-2 (PMC8295169; doi:10.1007/s00520-021-06059-2)
Supplement: Supplementary file 1 — (DOCX 88 kb) [file 520_2021_6059_MOESM1_ESM.docx]

**Supplementary information**

**Vinorelbine as substitute for vincristine in patients with diffuse large B cell lymphoma and vincristine induced neuropathy.**

Stefan Hatzl et al.

**Supplementary Table 1.** Baseline characteristics of the “Salzburg cohort” – Distribution by vincristine dose reduction (n=382).∙∙∙∙∙∙∙∙∙∙∙∙∙∙∙∙∙∙∙∙∙∙∙∙∙∙∙∙∙∙∙∙∙∙∙∙∙∙∙∙∙∙∙∙∙∙∙∙∙∙∙∙∙∙∙∙∙∙∙∙∙∙∙∙∙∙∙∙∙∙∙∙∙∙∙∙∙∙∙∙∙∙∙∙∙∙∙∙**p. 2**

**Supplementary Table 2.** Associations of vincristine dose reduction in the “Salzburg Cohort” and selected clinical co-variables with clinical outcomes in patients with DLBCL – uni- and multivariable Cox regression (n=382).∙∙∙∙∙∙∙∙∙∙∙∙∙∙∙∙∙∙∙∙∙∙∙∙∙∙∙∙∙∙∙∙∙∙∙∙∙∙∙∙∙∙∙∙∙∙∙∙∙∙∙∙∙∙∙∙∙∙∙∙∙∙∙∙∙∙∙∙∙∙∙∙∙∙∙∙∙∙∙∙∙∙∙∙**p. 3**

**Supplementary Table 3.** Oncologic outcomes in the “Graz Cohort” (n=605).∙∙∙∙∙∙∙∙∙∙∙∙∙∙∙∙∙∙∙∙∙p. 4

**Supplementary Table 4.** Univariable and multiple linear regression models of the number of first-line treatment cycles according to selected clinical co-variables.∙∙∙∙∙∙∙∙∙∙∙∙∙∙∙∙∙∙∙∙∙∙∙∙∙∙∙∙∙∙∙∙∙∙∙∙**p. 5**

**Supplementary Table 5.** Three measures of neuropathy at and after switch from vincristine to vinorelbine (n=199).∙∙∙∙∙∙∙∙∙∙∙∙∙∙∙∙∙∙∙∙∙∙∙∙∙∙∙∙∙∙∙∙∙∙∙∙∙∙∙∙∙∙∙∙∙∙∙∙∙∙∙∙∙∙∙∙∙∙∙∙∙∙∙∙∙∙∙∙∙∙∙∙∙∙∙∙∙∙∙∙∙∙∙∙∙∙∙∙∙∙∙∙∙∙∙∙∙∙∙∙∙∙∙∙∙∙∙∙∙∙∙∙**p. 6**

**Supplementary Table 6.** Associations of switching to vinorelbine and selected clinical co-variables with clinical outcomes in patients with DLBCL – uni- and multivariable Cox regression (n=605).∙∙∙∙∙∙∙∙∙∙∙∙∙∙∙∙∙∙∙∙∙∙∙∙∙∙∙∙∙∙∙∙∙∙∙∙∙∙∙∙∙∙∙∙∙∙∙∙∙∙∙∙∙∙∙∙∙∙∙∙∙∙∙∙∙∙∙∙∙∙∙∙∙∙∙∙∙∙∙∙∙∙∙∙∙∙∙∙∙∙∙∙∙∙∙∙∙∙∙∙∙∙∙∙∙∙∙∙∙∙∙∙∙∙**p. 7**

**Supplementary Table** **7.** Adverse events.∙∙∙∙∙∙∙∙∙∙∙∙∙∙∙∙∙∙∙∙∙∙∙∙∙∙∙∙∙∙∙∙∙∙∙∙∙∙∙∙∙∙∙∙∙∙∙∙∙∙∙∙∙∙∙∙∙∙∙∙∙∙∙∙∙∙∙∙∙∙∙∙∙∙∙∙∙∙**p. 8**

**Supplementary Table 8.** Exploratory analysis investigating the potential impact of switch time point on long-term oncologic outcomes.∙∙∙∙∙∙∙∙∙∙∙∙∙∙∙∙∙∙∙∙∙∙∙∙∙∙∙∙∙∙∙∙∙∙∙∙∙∙∙∙∙∙∙∙∙∙∙∙∙∙∙∙∙∙∙∙∙∙∙∙∙∙∙∙∙∙∙∙∙∙∙∙∙∙**p. 9**

**Supplementary Table legends.**∙∙∙∙∙∙∙∙∙∙∙∙∙∙∙∙∙∙∙∙∙∙∙∙∙∙∙∙∙∙∙∙∙∙∙∙∙∙∙∙∙∙∙∙∙∙∙∙∙∙∙∙∙∙∙∙∙∙∙∙∙∙∙∙∙∙∙∙∙∙∙∙∙∙∙∙∙∙∙∙∙∙∙∙∙∙∙∙∙∙∙∙**p.10**

**Supplementary Figure 1.** Time-to-event outcomes in the “Graz cohort” (n=605)∙∙∙∙∙∙∙∙∙∙∙∙∙**p. 12**

**Supplementary Table 1.**

| **Variable** |  | **N**  **(% miss.)** | **Overall**  **(n=382)** | **Full dose vincristine (R-CHOP)**  **(n=198)** | **Reduced dose vincristine (R-CHOP)**  **(n=184)** | **P-value** |
| --- | --- | --- | --- | --- | --- | --- |
|  |  |  |  |  |  |  |
| **Demographic variables** |  |  |  |  |  |  |
| Age (years) |  | 382 (0%) | 65 [63-66] | 58 [57-61] | 71 [69-73] | **0.009** |
| Female gender |  | 382 (0%) | 168 (44%) | 80 (40%) | 88 (48%) | 0.150 |
| ECOG (points) |  | 379 (1%) | 1 [0-1] | 1 [0-1] | 1 [0-1] | 0.199 |
| **Tumor characteristics** |  |  |  |  |  |  |
| Cell of origin: Non-GCB |  | 108 (71%) | 54 (50%) | 30 (52%) | 24 (50%) | 0.632 |
| Clinical stage: III-IV |  | 381 (0%) | 195 (51%) | 104 (52%) | 91 (49%) | 0.315 |
| Extranodal manifestation |  | 382 (0%) | 320 (53%) | 95 (47%) | 90 (49%) | 0.118 |
| **Risk stratification** |  |  |  |  |  |  |
| R-IPI (points) |  | 382 (0%) | 2 [2-3] | 2 [2-3] | 3 [2-3] | 0.354 |
| **Treatment characteristics** |  |  |  |  |  |  |
| Cycles of primary treatment |  | 382 (0%) | 6 [6-8] | 6 [6-8] | 6 [6-8] | 0.173 |
| Cumulative vincristine dose [mg] |  | 382 (0%) | 9.46 [9.06-9.86] | 11.50 [11.03-11.96] | 7.26 [6.78-7.76] | **<0.001** |
| Cumulative doxorubicin dose [mg] |  | 382 (0%) | 493 [476-511] | 515 [491-540] | 469 [443-495] | 0.479 |

**Supplementary Table 2.**

| **Endpoint** | **OS**  **Univariable** | **OS**  **Multivariable** | **PFS**  **Univariable** | **PFS**  **Multivariable** |
| --- | --- | --- | --- | --- |
|  |  |  |  |  |
| **Variable** | **HR (95%CI, p)** | **HR (95%CI, p)** | **HR (95%CI, p)** | **HR (95%CI, p)** |
| **Therapy** |  |  |  |  |
| Reduced dose vincristine (per 1mg decrease) | 1.60  (1.16-2.19, ***p<0.001***) | 1.54  (1.07-2.22, ***p=0.001***) | 1.47  (1.10-1.97, ***p<0.001***) | 1.45  (1.05-2.10, ***p=0.001***) |
| Reduced dose doxorubicin  (per 10mg decrease) | 1.26  (0.93-1.70, p=0.140) | 1.22  (0.93-1.60, p=0.159) | 1.21  (0.88-1.65, p=0.246) | 1.16  (0.87-1.64, p=0.345) |
|  |  |  |  |  |
| **R-IPI** |  |  |  |  |
| Very good | Ref. | Ref. | Ref. | Ref. |
| Good | 2.61  (1.68-4.05, ***p<0.001***) | 2.54  (1.57-3.82, ***p=0.001***) | 2.31  (1.21-4.43, ***p<0.001***) | 2.43  (1.27-4.63, ***p=0.002***) |
| Poor | 4.53  (2.62-7.83, ***p<0.001***) | 4.42  (2.55-7.64 ***p<0.001***) | 4.26  (3.26-5.26, ***p<0.001***) | 3.96  (2.93-4.99, ***p<0.001***) |
|  |  |  |  |  |
| **ECOG** |  |  |  |  |
| 0-2 | Ref. | Ref. | Ref. | Ref. |
| ≥3 | 2.75 (1.96–3.85, ***p<0.001***) | 1.46  (1.00–2.12, ***p=0.048***) | 2.20 (1.60–3.02, ***p<0.001***) | 1.40  (0.98–1.99, p=0.069) |

**Supplementary Table 3.**

| **Endpoint** |  | **1-year estimate (95%CI)** | **3-year estimate (95%CI)** | **5-year estimate (95%CI)** | **10-year estimate (95%CI)** |
| --- | --- | --- | --- | --- | --- |
|  |  |  |  |  |  |
| Overall  survival (OS) |  | 88%  (86-91) | 75%  (71-78) | 70%  (66-73) | 54%  (50-59) |
| Progression-free  survival (PFS) |  | 79%  (76-82) | 67%  (63-71) | 62%  (58-66) | 47%  (61-70) |
| Risk of primary progressive disease or relapse |  | 11%  (8-13) | 22%  (19-26) | 27%  (23-31) | 33%  (28-37) |
| Risk of death  from DLBLC |  | 9%  (7-12) | 19%  (16-23) | 22%  (18-25) | 26%  (22-30) |
| Risk of death  from other causes |  | 2%  (1-4) | 6%  (4-8) | 9%  (7-11) | 19%  (16-24) |

**Supplementary Table 4.**

| **Variable** |  | **Univariable linear regression models**  **(β (95%CI, p))** | **Multiple linear regression model**  **(β (95%CI, p))** |
| --- | --- | --- | --- |
|  |  |  |  |
| Switch to vinorelbine |  | 0.45  (0.16-0.74, **p=0.003**) | 0.25  (-0.01-0.52, p=0.063) |
| Treatment response |  | / | / |
| CR |  | Ref. | Ref. |
| PR |  | -0.80  (-1.27-(-0.33), **p=0.001**) | -0.78  (-1.25-(-0.31), **p=0.001**) |
| PD |  | -2.53  (-2.95-(-2.12), **p<0.001**) | -2.48  (-2.90-(-2.07), **p<0.001**) |
| NCCN-IPI  (per 1 point increase) |  | -0.15  (-0.23-(-0.07), **p<0.001**) | N/A |

**Supplementary Table 5.**

| **Neuropathy measure** | **At switch to vinorelbine (Mean±SD)** | **2 months after switch to vinorelbine (Mean±SD)** | **Mean difference**  **(95%CI)** | **P-Value** |
| --- | --- | --- | --- | --- |
|  |  |  |  |  |
| NCI CTC AE (grades) | 1.8±0.6 | 0.7±0.8 | -1.1 (-1.22-(-1.0)) | **<0.0001** |
| cTNS grade (grades) | 2.0±0.7 | 1.4±0.5 | -0.6 (-0.7-(-0.5)) | **<0.0001** |
| cTNS score (points) | 10.5±4.8 | 6.8±4.0 | -3.7 (-4.4-(-3.0)) | **<0.0001** |

**Supplementary Table 6.**

| **Endpoint** | **OS**  **Univariable** | **OS**  **Multivariable** | **PFS**  **Univariable** | **PFS**  **Multivariable** | **DLBCL mortality**  **Univariable** | **DLBCL mortality Multivariable** |
| --- | --- | --- | --- | --- | --- | --- |
|  |  |  |  |  |  |  |
| **Variable** | **HR (95%CI, p)** | **HR (95%CI, p)** | **HR (95%CI, p)** | **HR (95%CI, p)** | **HR (95%CI, p)** | **HR (95%CI, p)** |
| Switch to  vinorelbine | 0.80  (0.61-0.99, **p=0.030**) | 0.65  (0.45-0.94,  **p=0.021**) | 0.81  (0.63-0.98, **p=0.037**) | 0.71  (0.51-0.99,  **p=0.044**) | 0.67  (0.46-0.98, **p=0.038**) | 0.62  (0.39-0.97,  **p=0.038**) |
| NCCN-IPI  (per point increase) | 1.48  (1.38-1.59, **p<0.001**) | 1.49  (1.35-1.64,  **p<0.001**) | 1.39  (1.30-1.49, **p<0.001**) | 1.38  (1.26-1.50,  **p<0.001**) | 1.45  (1.32-1.60, **p<0.001**) | 1.48  (1.31-1.66,  **p<0.001**) |
| R-IPI  (per point increase) | 1.57 (1.42-1.75, **p<0.001**) | N/A | 1.51  (1.37-1.66, **p<0.001**) | N/A | 1.59  (1.39-1.83, **p<0.001**) | N/A |
| CNS-IPI  (per point increase) | 1.45 (1.29-1.63, **p<0.001**) | N/A | 1.47  (1.32-1.63, **p<0.001**) | N/A | 1.61  (1.39-1.87, **p<0.001**) | N/A |
| DEL  (per point increase) | 1.59  (1.26-2.02, **p<0.001**) | 1.55  (1.22-1.96,  **p<0.001**) | 1.57  (1.27-1.95, **p<0.001**) | 1.51  (1.22-1.87,  **p<0.001**) | 1.94  (1.44-2.60, **p<0.001**) | 1.87  (1.39-2.51,  **p<0.001**) |
| COO: Non-GCB | 1.78 (1.36-2.33, **p<0.001**) | 1.20  (0.87-1.66,  p=0.260) | 1.77  (1.39-2.26, **p<0.001**) | 1.13  (0.84-1.51,  p=0.429) | 1.99  (1.41-2.83, **p<0.001**) | 1.29  (0.86-1.93,  p=0.219) |

**Supplementary Table 7.**

| **Adverse Event** | **N (% miss.)** | **R-CHOP (n=406)** | **Vino-R-CAP**  **(n=199)** | **p-value** |
| --- | --- | --- | --- | --- |
| Neutropenia (≥grade 3) | 605 (0%) | 344 (57%) | 122 (61%) | 0.052 |
| Thrombocytopenia (≥grade 3) | 605 (0%) | 8 (2%) | 2 (1%) | 0.870 |
| Febrile neutropenia | 605 (0%) | 9 (2%) | 5 (2%) | 0.976 |
| Infection (any grade) | 605 (0%) | 195 (48%) | 92(46%) | 0.256 |
| Cardiac toxicity (≥grade 3) | 436 (28%) | 25 (6%) | 10 (5%) | 0.674 |
| Renal toxicity (≥grade 3) | 547 (5%) | 3 (1%) | 0 (0%) | 0.787 |
| Liver toxicity (≥grade 3) | 547 (5%) | 7 (2%) | 2 (1%) | 0.547 |

**Supplementary Table 8.**

| **Endpoint** | **Overall**  **survival** | **Progression-free survival** | **DLBCL-related mortality** |
| --- | --- | --- | --- |
|  |  |  |  |
| **Switch subgroup** | **HR**  **(95%CI, p)** | **HR**  **(95%CI, p)** | **HR**  **(95%CI, p)** |
| No switch to  vinorelbine | Ref. | Ref. | Ref. |
| Switch to vinorelbine during or before 3^rd^ cycle | 0.83  (0.60-1.14, p=0.247) | 0.86  (0.64-1.14) | 0.76  (0.51-1.15, p=0.198) |
| Switch to vinorelbine after 3^rd^ cycle | 0.60  (0.36-1.00, p=0.052) | 0.55  (0.34-0.90, p=0.017) | 0.48  (0.23-1.00, p=0.048) |

**Supplementary Table legends.**

**Supplementary Table 1. Baseline characteristics of the “Salzburg cohort” – Distribution by vincristine dose reduction (n=382).** N (%miss.) denotes the number of patients with fully observed variable (% missing). P-values are from ranksum-tests, χ^2^-tests, and Fisher’s exact tests, as appropriate, Abbreviations: GCB – Germinal center B-cell, R-IPI – Revised International Prognostic Index, DLBCL – Diffuse large B-cell lymphoma.

**Supplementary Table 2. Associations of vincristine dose reduction in the “Salzburg Cohort” and selected clinical co-variables with clinical outcomes in patients with DLBCL – uni- and multivariable Cox regression (n=382).** Abbreviations: OS – overall survival, PFS – progression-free survival, DLBCL – diffuse large B-cell lymphoma, HR – hazard ratio, 95%CI – 95% confidence interval, R-IPI – Revised International Prognostic Index, ECOG – Eastern Cooperative Oncology Group.

**Supplementary Table 3. Oncologic outcomes in the “Graz Cohort” (n=605).** OS and PFS were estimated with Kaplan-Meier estimators, whereas risks of the other endpoints were computed with competing risk cumulative incidence estimators. Abbreviations: 95%CI – 95% confidence interval, DLBCL – diffuse large B-cell lymphoma. Abbreviations: OS – overall survival, PFS – progression-free survival, DLBCL – diffuse large B-cell lymphoma.

**Supplementary Table 4. Univariable and multiple linear regression models of the number of first-line treatment cycles according to selected clinical co-variables.** Primary estimates are the β coefficient, representing the change in the number of first-line treatment cycles per one unit increase in the predictor variable (e.g. in univariable linear regression, patients who were switched to vinorelbine received an average of 0.45 treatment cycles more than those who were not switched, respectively). Abbreviations: β – regression coefficient, 95%CI – 95% confidence interval, p – Wald-test p-value, CR – complete remission, PR – partial remission, PD – progressive disease, NCCN-IPI – National Comprehensive Cancer Network International Prognostic Index, Ref. – reference category, N/A – not applicable.

**Supplementary Table 5. Three measures of neuropathy at and after switch from vincristine to vinorelbine (n=199).** Data are means ± standard deviation, p-values are from paired t-tests. Abbreviations: SD – standard deviation, 95%CI – 95% confidence interval, NCI CTC AE – national cancer institute common terminology criteria for adverse events, cTNS – clinical total neuropathy score.

**Supplementary Table 6. Associations of switching to vinorelbine and selected clinical co-variables with clinical outcomes in patients with DLBCL – uni- and multivariable Cox regression (n=605).** Abbreviations: OS – overall survival, PFS – progression-free survival, DLBCL – diffuse large B-cell lymphoma, HR – hazard ratio, 95%CI – 95% confidence interval, p – Wald test p-value, N/A – not applicable, NCCN-IPI – National Comprehensive Cancer Network International Prognostic Index, CNS-IPI – Central Nervous System International Prognostic Index, DEL – Double expressor lymphoma biology (point-based system according to Green et al.)^1^, COO – Cell of origin, GCB – Germinal center B-cell.

**Supplementary Table 7. Adverse events.**

An adverse event was defined as adverse change from patient’s baseline condition, whether it was related to treatment or not. Each event was graded according to National Cancer Institute Common Terminology Criteria for Adverse Events grading system version 3; R-CHOP denotes rituximab, cyclophosphamide, doxorubicin, vincristine, prednisone; c-reactive protein (CRP) increase of 10 fold of baseline was considered as infection; Vino-R-CAP denotes rituximab, cyclophosphamide, doxorubicin, vinorelbine, prednisone.

P-values are from, χ^2^-tests and Fisher’s exact tests, as appropriate.

**Supplementary Table 8. Exploratory analysis investigating the potential impact of switch time point on long-term oncologic outcomes.** Patients were empirically categorized into “no switch”, “switch to vinorelbine during or before third cycle”, and “switch to vinorelbine after third cycle”. P-value was calculated with Wald-test. Abbreviations: DLBCL – diffuse large B-cell lymphoma, HR – hazard ratio, 95%CI – 95% confidence interval, Ref. – Reference category.

**Supplementary Figure 1.**

**Supplementary Figure 1. Time-to-event outcomes in the “Graz cohort” (n=605).** Overall survival and progression-free survival curves were estimated with Kaplan-Meier estimators, whereas curves of the other endpoints were computed with competing risk cumulative incidence estimators.

**REFERENCES**

1. Green TM, Young KH, Visco C, et al: Immunohistochemical double-hit score is a strong predictor of outcome in patients with diffuse large B-cell lymphoma treated with rituximab plus cyclophosphamide, doxorubicin, vincristine, and prednisone. J Clin Oncol 30:3460-3467, 2012
